# Gait stability in virtual reality: effects of VR display modality in the presence of visual perturbations

**DOI:** 10.1186/s12984-025-01558-3

**Published:** 2025-02-21

**Authors:** Elizabeth B. Wilson, J. Stephen Bergquist, W. Geoffrey Wright, Daniel A. Jacobs

**Affiliations:** 1 Mechanical Engineering, University of Michigan, Ann Arbor, MI USA; 2 Health and Rehabilitation Sciences, Temple University, Philadelphia, PA USA; 3 Mechanical Engineering, Temple University, Philadelphia, PA USA

**Keywords:** Virtual reality (VR), Locomotion, Stability, Visual perturbations, Visuomotor processing

## Abstract

**Purpose:**

Virtual reality (VR) has emerged as a pivotal tool for studying balance and postural control mechanisms, leveraging unpredictable visual disturbances that dynamically challenge visuomotor processing. However, the quantity and quality of information available in the visual field may differ between VR systems, potentially introducing conflict with the intended perturbation inputs. Consequently, the extent to which a VR system used in a visual perturbation paradigm influences its ability to elicit compensatory gait behaviors remains unclear. Here we investigate the impact of (1) VR display modality and (2) the direction of visual perturbations on spatiotemporal gait parameters and measures of stability in VR.

**Methods:**

Participants were tasked with maintaining steady-state walking on a self-paced treadmill while viewing a VR scene presented in either a rear-projection curved screen immersive room (IR) or a head-mounted display (HMD). During trials with augmented visual perturbations, pseudorandom oscillations were combined with forward walking speed either in the anterior-posterior (AP), or medio-lateral (ML) direction. Linear mixed-effects models were used to analyze the impact of VR display type and visual perturbations on spatiotemporal gait parameters, stability measures, and joint kinematics.

**Results:**

For self-paced walking in matched VR optic flow, we found that the HMD increased the variability of several parameters related to walking speed control, but did not significantly impact any gait parameter average values. Superimposing visual perturbations along the ML axis increased gait variability and decreased walking stability in both VR systems, but the perturbations had stronger effects if presented in the HMD.

**Conclusion:**

Together, these findings suggest that portable light-weight HMD systems can provide affordable, reliable tools for studying and training balance control and locomotion.

**Supplementary Information:**

The online version contains supplementary material available at 10.1186/s12984-025-01558-3.

## Background

Virtual reality (VR) has been increasingly leveraged for the detection [1, 2] and rehabilitation [3–6] of balance deficits as an alternative to traditional approaches. Musculoskeletal degeneration and sensory losses disproportionately impact older adults [7, 8] and several clinical populations [9–12], in turn, degrading their functional capacity for maintaining walking stability. In combination with external factors encountered while navigating one’s environment, these unstable gait patterns increase fall risk under perturbing conditions [13]. Therefore, to mitigate the negative health impacts associated with falling, it becomes critical to develop screening and intervention strategies that can address nuanced biomechanical responses to balance threats. Advantageous for constructing ecologically valid assessments, VR can simulate destabilizing scenarios that may otherwise be impossible to replicate in a traditional testing environment.

VR-based paradigms provide an effective tool for challenging dynamic stability during active balance control by allowing the systematic manipulation of visual input [14–16]. Locomotion is governed by cortical integration of sensory inputs from vestibular, proprioceptive, and visual processes. Introducing discrete [17, 18] or continuous [19, 20] perturbations to the visual field decreases the reliability of the input, requiring neural modulation to reweight the relevant sensory information. Failure to habituate to or downweight discordant visual stimuli can significantly impair the ability to establish an accurate perception of spatial orientation and adjust to external disturbances accordingly based on these internal models [21].

In response to visual perturbations, locomotor adaptations indicative of a cautious gait strategy have been observed while navigating virtual environments [22–25]. Namely, the introduction of visual discordance in a virtual environment is typically associated with shorter, slower, wider, and more variable steps—hallmarks of a strategy to maintain or restore gait stability [26, 27]—when compared to walking in the real-world. The extent of these locomotor effects is anisotropic, governed by the directional axis of the perturbation. Perturbations orthogonal to the forward gait velocity (i.e. medio-lateral (ML) perturbations) induce more pronounced impacts on the spatio-temporal gait parameters than anterior–posterior (AP) perturbations applied in parallel to forward walking speed [22]. It is suggested that ML oscillations have a more profound effect on locomotion due to the differing control mechanisms required to maintain stability in each plane. Stability in the direction of progression benefits from the body’s intrinsic passive dynamics, whereas stability in the ML plane requires the engagement of active neural control through sensory feedback [15]. Consequently, the reliance on visual information in the lateral plane amplifies the vulnerability to disturbances. Moreover, ML oscillations are uncorrelated with forward motion, thereby making them more detectable to visual perceptual processes [28, 29].

Two distinct types of VR systems harnessed for gait assessment are head-mounted displays (HMDs) and projection-based immersive rooms (IRs). Each offers a unique display experience that could potentially influence locomotor behaviors [30]. A number of hardware properties that vary between HMD and IR systems have been shown to distort perception in a virtual environment including field of view [31], focal distance [32], and resolution [33]. Ergonomic factors, namely the increased external weight from the HMD, may also impact user behaviors, particularly during tasks that require extensive movement [34]. Further, beyond differences in the presented images, IRs might contain earth-fixed anchoring cues from the external environment, such as the physical boundaries of the projector screen [32] and body representation [35–37], enabling users to leverage real-world information for internal locomotor models. Consequently due to these, or other hardware differences, previous research has demonstrated that HMDs are more effective in inducing changes to spatiotemporal gait parameters in response to optic flow speed manipulations when compared to an immersive room [38]. Despite these advancements, little is currently known about whether differences in the visual information provided by each VR system similarly affect balance responses to visual perturbations. Specifically, the relative abilities of both VR systems to provide controlled visual stimulation robust enough to elicit meaningful changes in stability measures remains underexplored.

Understanding the impact of display type on gait control and perturbation responses may assist in refining experimental protocols, thus improving the sensitivity of VR-based studies to capture subtle, yet fundamental differences in balance adaptations. Therefore, the objectives of our study are twofold: to investigate the impact of VR display type on normative gait behaviors, and to explore how the directional axis of visual perturbations alters spatiotemporal gait parameters and measures of stability in each VR display type. We hypothesized that (1) unperturbed, velocity-matched (isometric) optic flow will lead to more pronounced deviations from real-world treadmill walking patterns when presented in the HMD compared to the IR, (2) the directional axis of visual perturbations will differentially affect spatiotemporal gait parameters and stability measures, with ML perturbations having a greater impact on balance control compared to AP, and (3) perturbation-induced changes to stability will be more prominent in the HMD than in the IR.

## Methods

**Fig. 1:**
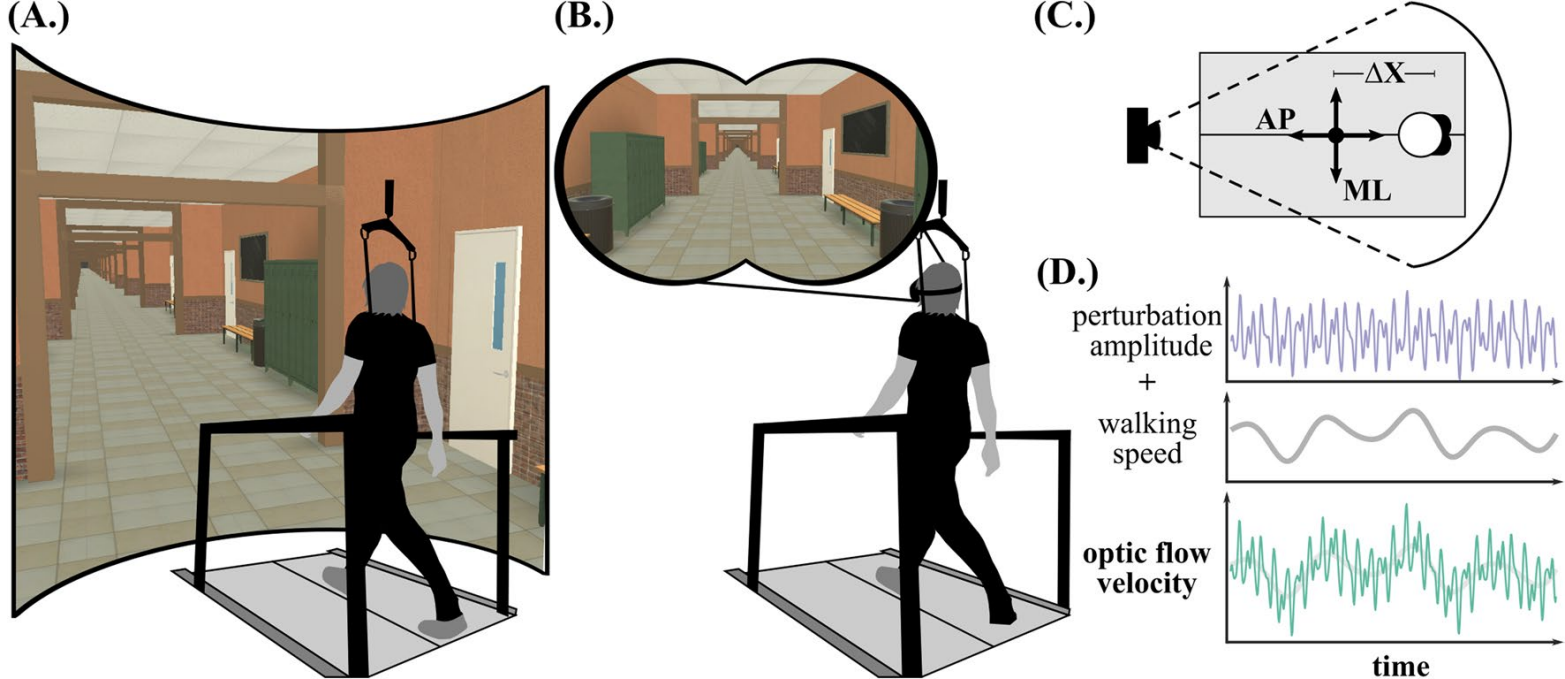
Third-person perspective for the virtual environment presented in (**A**) an integrated VR projection dome (IR) and (**B**) a head-mounted display (HMD). **C** Top-down view of the self-paced treadmill setup showing the anterior–posterior (AP) and medial–lateral (ML) axes, along which the visual perturbations were applied. $\Delta$X represents the participant’s displacement along the treadmill toward the projection screen, used to update self-paced belt speed. **D** Visualization of the optic flow stimulus applied during AP perturbation conditions. Controlled visual noise, dictated by a pseudorandom sum of sines function, is superimposed onto the participant’s current walking speed to introduce dynamic variations in perceived forward motion

### Participants

16 unimpaired young adults participated in the experiment (9 females; age 30.2 ± 6.7 years; height 173.6 ± 8.0 cm; mass 70.6 ± 14.6 kg). Participants identified themselves as having at least 20/40 vision, either naturally or with correction. Participants with a self-reported history of visually induced motion sickness were excluded from this study, and no adverse effects (e.g. dizziness, light-headedness, or nausea) were reported throughout VR immersion. Prior to beginning the experiment, participants provided informed written consent in accordance with the Temple University Institutional Review Board approved protocol (#29507).

### Equipment

Participants performed the experiment on an instrumented treadmill (Bertec, Ohio, USA) operating in a self-paced walking mode (SPT). The treadmill control algorithm dynamically adjusted belt speed based on the participant’s estimated pelvis displacement to maintain their position near the center of the belt. Pelvis displacement was estimated in real-time using motion capture data as the average location of four anatomical landmarks (bilateral anterior and posterior superior iliac spines). The following equation was used to calculate commanded accelerations for the treadmill motors ($a_{treadmill}$):

1 $$\begin{aligned} a_{treadmill} = K_{p}{\Delta }X + K_{d}\frac{\Delta X}{\Delta t} \end{aligned}$$

where ${K_p}$ and ${K_d}$ are tuned proportional and derivative gain values respectively, $\Delta$X is pelvis displacement from the center of the treadmill, and $\frac{\Delta X}{\Delta t}$ is the relative pelvis velocity [39]. A 9-camera motion capture system (Vicon, Oxfordshire, United Kingdom) tracked 41 passive markers on the lower extremities, trunk, and head at 1000 Hz, providing the necessary data for pelvis position estimation and treadmill speed adjustments.

Our custom developed virtual environment (Unity Technologies, California, USA) depicted an infinitely-repeating school hallway scene. Depth cues were simulated using 3D objects (e.g. lockers, benches, chalkboards) scaled to real-world dimensions. Participants viewed the scene from a first-person perspective without rendered avatar feedback regarding body position. Forward translation of the scene was coupled with real-time SPT estimations to establish an optic flow pattern isometrically matched to walking speed as previously described by the authors [40]. The immersive room (IR) conditions used an integrated VR projection dome (Immersive Labs; Bertec Corporation, Ohio, USA) to display the virtual hallway environment (Fig. 1A), while participants wore a VR headset (Oculus Quest; Facebook Technologies, CA, USA) for head-mounted display (HMD) conditions (Fig. 1B).

Lateral handrails and a suspended harness were employed to reduce fall risk during all conditions, though participants were discouraged from touching the handrails unless safety was an immediate concern. The suspended harness engaged only in the event of a fall and did not otherwise provide any meaningful stabilizing force or sensory feedback.

### Experimental protocol

**Fig. 2:**
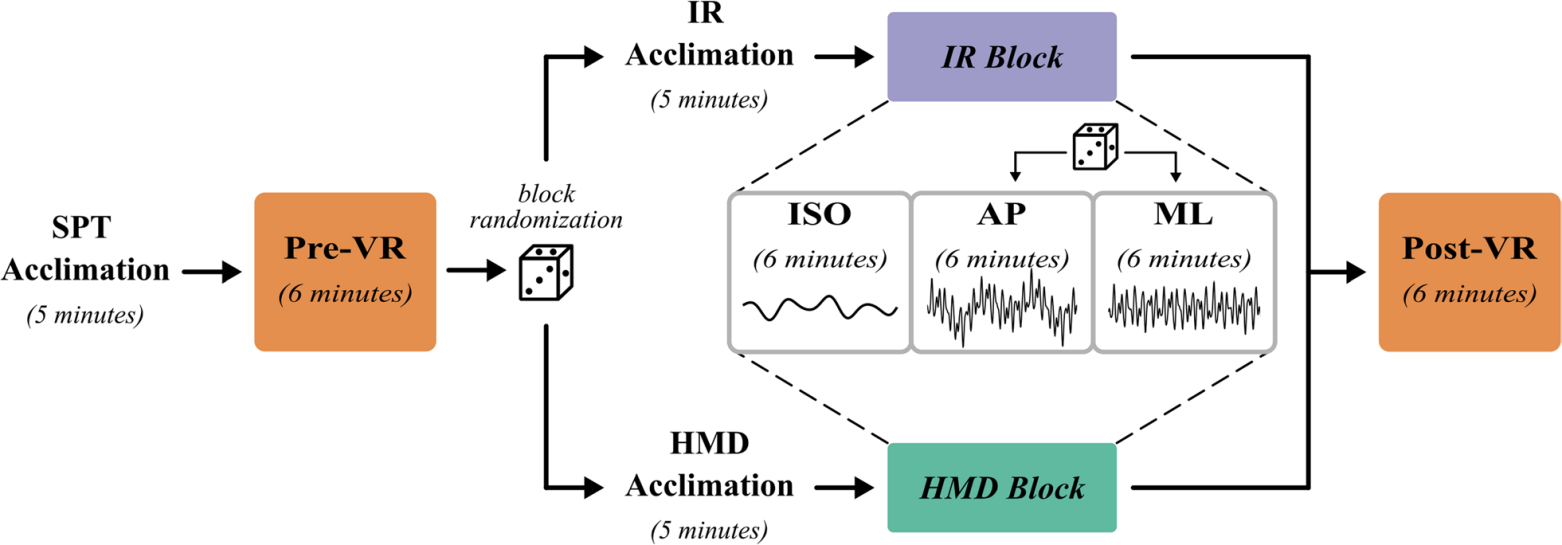
Schematic representation of the experimental protocol. The first and final walking tasks were performed without displaying the virtual environment (*Pre-VR* and *Post-VR* conditions). VR trials with matched optic flow (*ISO*) and perturbations along each axis (*AP* and *ML*) were conducted in both the HMD and IR

Participants completed eight 6-min self-paced walking trials in the following sequence: (1) a baseline trial conducted visual perturbations or forward optic flow (Pre-VR), (2) the first VR experimental block of three trials, conducted either with the HMD or IR presentation modality, (3) a second VR block of three trials, using the remaining presentation modality, and (4) a post-test trial (Post-VR), identical to the baseline (Fig. 2). Each VR block was comprised of three trials corresponding to the visual perturbation conditions: isometric (ISO), anterior-posterior (AP), and mediolateral (ML). Each participant completed all eight conditions (Pre-VR, IR-ISO, IR-AP, IR-ML, HMD-ISO, HMD-AP, HMD-ML, Post-VR), with each condition conducted exactly once per participant. In both VR blocks, the ISO condition was always presented first, then followed by the visual perturbation trials. To control for learning effects, the order of IR and HMD trial blocks was counterbalanced pseudorandomly across participants, as was the order of AP and ML perturbation trials within each block.

Baseline walking trials for each VR block (IR-ISO, HMD-ISO) featured isometric forward optic flow driven solely by participants’ walking speed, without additional visual perturbations. In perturbed conditions (IR-ML, IR-AP, HMD-ML, HMD-AP), continuous visual oscillations were either superimposed over walking speed in AP direction or in the ML direction (Fig. 1C). During AP perturbations, visual oscillations fluctuated between velocities faster and slower than isometric, occasionally causing the scene to move backward relative to the participant’s forward motion (Fig. 1D). During ML perturbations, forward motion of the virtual scene remained isometrically tied to walking speed while the focal perspective shifted from side-to-side within the lateral bounds of the virtual hallway. Perturbation distance was dictated by the following non-repeating pseudorandom sum of sines equation:

2 $$\begin{aligned} D(t) &= A[sin(0.16*2{\pi }t) + 0.8 sin(0.21*2{\pi }t) \\ &\quad+1.4sin(0.24*2{\pi }t) + 0.5sin(0.49*2{\pi }t)] \end{aligned}$$

where *A* is a scaling factor (0.125), *t* is time, and each of the constants (0.16, 0.21, 0.24 and 0.49 Hz) reflect the summed oscillation frequencies [19]. Scaling factor magnitude, *A*, was selected by constraining the perturbation distance to ensure that the visual field would not exceed lateral virtual hallway boundaries at amplitude extremes. Following previously established guidelines [19], pilot testing was conducted to verify that the perturbation scaling elicited visibly perceptible changes to walking behavior, without inducing falls or nausea.

During trials without augmented dynamic visual information (Pre-VR, Post-VR), participants viewed static visual objects designed to provide analogous peripheral and focal visual cues to those in the VR trial blocks. Instead of the hallway scene, a focal point was provided via a simple 1 m${^2}$ image of a yellow *X* aligned with the custom scene’s vanishing point. Virtual projections of low-visual density asphalt walls, with dimensions matching those of the hallway scene, provided peripheral information comparable to the VR trials, but without any augmented motion cues. These visual assets were layered over a generic horizon backdrop, filling the remaining visual field. While the projection was delivered using the IR system, its primary role was limited to providing this fixation target and peripheral visual information.

Participants were given scripted instructions before every trial to walk in the center of the treadmill, focus their gaze on the fixation target, and avoid touching the railings unless needed to stay upright. Participants were provided a fixation target to encourage a steady gaze and minimize excessive head motion, though head movements were not explicitly tracked during the trials. Prior to the baseline Pre-VR trial and each of the VR blocks, participants underwent a 5-min guided orientation to familiarize them with the respective treadmill and VR systems. One-minute standing breaks were provided between all trials, in addition to a 5-min seated break between the two VR blocks.

### Data and statistical analysis

The final 450 steps taken by participants in each condition were included for analysis, with the initial ramp-up stage of gait initiation excluded to ensure steady-state walking.

#### Spatiotemporal gait parameters

Spatiotemporal gait parameters—walking speed (WS), step length (SL), and step time (ST)—were calculated between subsequent heelstrikes on contralateral sides. Mean values and corresponding standard deviations (SD), used to represent variability measures—walking speed variability (WSV), step length variability (SLV), and step time variability (STV)—were determined over all included steps in each condition. SL was calculated using a kinematic correction for treadmill belt translation as previously described by the authors [41]. Briefly, this method sums the additional distance traveled on a moving surface during the push-off phase of gait with the difference in AP position of the bilateral calcaneus markers at heel strike.

#### Stability outcomes

Margin of stability (MOS) in the medial direction was bilaterally assessed during the single-support stance phase of gait. A minimum value for MOS, specified by the horizontal distance between the extrapolated center of mass (XCOM) and the closest boundary of the base of support (BOS), was determined for each step. Markers on the 1st and 5th metatarsi demarcated the BOS boundaries for each respective foot during single-support. XCOM was calculated using the following equation:

3 $$\begin{aligned} XCOM = P_{COM} + \frac{V_{COM}}{\sqrt{g/L}} \end{aligned}$$

4 $$\begin{aligned} MOS = BOS - XCOM \end{aligned}$$

where ${P_{COM}}$ and ${V_{COM}}$ are the position and velocity respectively for the estimated COM, *g* is the gravitational constant ($9.81 \, {\text {m/s}}^2$), and *L* is the length of the participant’s leg as measured by the average height of the two greater trochanter markers [42]. Negative values for MOS were defined by the XCOM exceeding BOS boundaries and are indicative of mechanical instability.

Step width means (SW) and corresponding variability measures (SWV) were calculated using successive heelstrikes as previously described for the additional spatiotemporal gait metrics. The lateral distance between calcaneous markers at heelstrike defined SW for each step.

#### Joint kinematics

Ankle and knee angles in the sagittal plane, along with hip angles in both the frontal and sagittal plane were calculated to assess joint kinematics. Inverse kinematic analysis was performed using OpenSim’s 4.5 API. The native Gait-2354 [43] lower limb model (19 degrees of freedom) was scaled to participant specific geometry based on a static standing trial. Output kinematic data was low-pass filtered at 15 Hz using a second order butterworth filter. Resultant angle trajectories were time normalized over gait cycles for contralateral sides. Measured parameters included peak angle and range of motion (ROM) for each joint. The mean and SD of the dependent kinematic variables were quantified across all gait cycles in each condition (Table 1).

**Table 1:** Spatiotemporal parameters

Condition	Mean			Variability		
	Velocity (m/s)	Step length (m)	Step time (s)	Velocity SD (m/s)	Step length SD (m)	Step time SD (s)
Pre-VR	1.08$\,\pm \,$0.24	0.63$\,\pm \,$0.09	0.59$\,\pm \,$0.07	0.095$\,\pm \,$0.029	0.043$\,\pm \,$0.012	0.022$\,\pm \,$0.007
IR-ISO	1.17$\,\pm \,$0.26	0.65$\,\pm \,$0.12	0.57$\,\pm \,$0.05	0.091$\,\pm \,$0.007	0.038$\,\pm \,$0.013	0.022$\,\pm \,$0.008
IR-AP	1.25$\,\pm \,$0.21	0.69$\,\pm \,$0.09	0.55$\,\pm \,$0.04	0.094$\,\pm \,$0.024	0.036$\,\pm \,$0.010	0.025$\,\pm \,$0.008
IR-ML	1.23$\,\pm \,$0.23	0.66$\,\pm \,$0.10	0.54$\,\pm \,$0.04	0.110$\,\pm \,$0.029	0.048$\,\pm \,$0.016	0.026$\,\pm \,$0.010
HMD-ISO	1.18$\,\pm \,$0.25	0.65$\,\pm \,$0.11	0.56$\,\pm \,$0.05	0.148$\,\pm \,$0.067	0.057$\,\pm \,$0.023	0.036$\,\pm \,$0.022
HMD-AP	1.21$\,\pm \,$0.29	0.66$\,\pm \,$0.13	0.56$\,\pm \,$0.04	0.140$\,\pm \,$0.059	0.055$\,\pm \,$0.025	0.031$\,\pm \,$0.015
HMD-ML	1.17$\,\pm \,$0.27	0.60$\,\pm \,$0.12	0.53$\,\pm \,$0.01	0.202$\,\pm \,$0.057	0.083$\,\pm \,$0.023	0.046$\,\pm \,$0.019
Post-VR	1.39$\,\pm \,$0.23	0.73$\,\pm \,$0.07	0.53$\,\pm \,$0.05	0.082$\,\pm \,$0.034	0.031$\,\pm \,$0.012	0.017$\,\pm \,$0.007

**Table 2:** Stability outcomes

Condition	Mean		Variability	
	Step width (cm)	MOS (cm)	Step width SD (cm)	MOS SD (cm)
Pre-VR	13.18$\,\pm \,$4.07	−5.27$\,\pm \,$3.20	2.40$\,\pm \,$0.64	1.81$\,\pm \,$0.52
IR-ISO	13.05$\,\pm \,$2.75	−4.94$\,\pm \,$3.04	2.84$\,\pm \,$0.67	2.61$\,\pm \,$1.48
IR-AP	12.13$\,\pm \,$2.78	−4.92$\,\pm \,$2.46	3.10$\,\pm \,$0.65	2.35$\,\pm \,$0.52
IR-ML	13.65$\,\pm \,$4.21	−6.05$\,\pm \,$3.19	5.40$\,\pm \,$1.42	4.24$\,\pm \,$0.93
HMD-ISO	12.81$\,\pm \,$3.26	−4.68$\,\pm \,$3.18	3.07$\,\pm \,$0.75	2.58$\,\pm \,$0.78
HMD-AP	12.59$\,\pm \,$3.31	−5.07$\,\pm \,$2.50	3.23$\,\pm \,$0.65	2.53$\,\pm \,$0.47
HMD-ML	14.58$\,\pm \,$3.97	−7.35$\,\pm \,$2.55	7.18$\,\pm \,$2.09	5.69$\,\pm \,$1.32
Post-VR	11.64$\,\pm \,$2.49	−5.03$\,\pm \,$2.30	3.01$\,\pm \,$0.71	2.27$\,\pm \,$0.55

**Table 3:** Joint kinematics

Condition	ROM ($^{\circ }$)				ROM SD ($^{\circ }$)			
	Hip add.	Hip flex.	Knee flex.	Ankle flex.	Hip add.	Hip flex.	Knee flex.	Ankle flex.
Pre-VR	12.81$\,\pm \,$3.19	40.49$\,\pm \,$5.24	66.98$\,\pm \,$5.17	30.88$\,\pm \,$5.11	1.48$\,\pm \,$0.59	2.11$\,\pm \,$0.57	2.90$\,\pm \,$0.94	3.71$\,\pm \,$1.71
IR-ISO	13.43$\,\pm \,$3.49	42.16$\,\pm \,$5.93	68.45$\,\pm \,$5.26	31.88$\,\pm \,$5.42	1.63$\,\pm \,$0.64	1.92$\,\pm \,$0.63	2.64$\,\pm \,$1.28	3.56$\,\pm \,$1.01
IR-AP	14.06$\,\pm \,$3.72	43.43$\,\pm \,$4.99	69.63$\,\pm \,$4.84	32.44$\,\pm \,$4.95	1.50$\,\pm \,$0.52	1.79$\,\pm \,$0.45	2.48$\,\pm \,$0.76	3.38$\,\pm \,$0.89
IR-ML	13.44$\,\pm \,$3.39	42.62$\,\pm \,$5.30	68.67$\,\pm \,$5.28	31.48$\,\pm \,$5.35	1.81$\,\pm \,$0.50	2.27$\,\pm \,$0.67	2.97$\,\pm \,$0.96	3.74$\,\pm \,$0.89
HMD-ISO	13.88$\,\pm \,$3.80	42.20$\,\pm \,$6.39	68.03$\,\pm \,$6.15	30.54$\,\pm \,$5.42	1.69$\,\pm \,$0.51	2.16$\,\pm \,$0.56	3.20$\,\pm \,$1.22	3.88$\,\pm \,$1.40
HMD-AP	13.83$\,\pm \,$3.93	42.56$\,\pm \,$7.00	67.84$\,\pm \,$6.93	31.62$\,\pm \,$6.16	1.68$\,\pm \,$0.52	2.22$\,\pm \,$1.16	3.02$\,\pm \,$2.14	3.89$\,\pm \,$1.23
HMD-ML	12.70$\,\pm \,$3.01	40.41$\,\pm \,$6.30	65.42$\,\pm \,$6.82	28.13$\,\pm \,$4.69	2.26$\,\pm \,$0.58	3.47$\,\pm \,$0.91	4.59$\,\pm \,$1.69	4.95$\,\pm \,$1.43
Post-VR	14.48$\,\pm \,$3.14	44.88$\,\pm \,$4.06	68.83$\,\pm \,$5.53	32.78$\,\pm \,$4.06	1.60$\,\pm \,$0.54	1.87$\,\pm \,$0.57	2.17$\,\pm \,$0.72	3.01$\,\pm \,$1.10

Condition	Peak angle ($^{\circ }$)				Peak angle SD ($^{\circ }$)			
	Hip add.	Hip flex.	Knee flex.	Ankle flex.	Hip add.	Hip flex.	Knee flex.	Ankle flex.
Pre-VR	7.12$\,\pm \,$2.18	24.06$\,\pm \,$9.35	69.28$\,\pm \,$3.66	20.24$\,\pm \,$3.81	2.41$\,\pm \,$2.03	1.51$\,\pm \,$0.63	2.40$\,\pm \,$0.79	2.85$\,\pm \,$1.86
IR-ISO	7.37$\,\pm \,$2.32	24.72$\,\pm \,$10.01	70.14$\,\pm \,$4.28	20.76$\,\pm \,$4.37	1.93$\,\pm \,$0.99	1.47$\,\pm \,$0.53	2.14$\,\pm \,$1.09	2.79$\,\pm \,$0.93
IR-AP	7.78$\,\pm \,$2.23	25.25$\,\pm \,$9.83	71.22$\,\pm \,$3.55	21.29$\,\pm \,$4.64	1.95$\,\pm \,$1.01	1.45$\,\pm \,$0.45	1.82$\,\pm \,$0.38	2.80$\,\pm \,$0.88
IR-ML	7.16$\,\pm \,$2.51	25.25$\,\pm \,$9.90	70.82$\,\pm \,$3.88	20.95$\,\pm \,$4.60	2.24$\,\pm \,$0.87	1.66$\,\pm \,$0.47	2.24$\,\pm \,$0.71	3.19$\,\pm \,$0.89
HMD-ISO	7.57$\,\pm \,$2.27	24.64$\,\pm \,$9.21	70.35$\,\pm \,$4.43	20.62$\,\pm \,$4.55	2.52$\,\pm \,$1.93	1.67$\,\pm \,$0.54	2.34$\,\pm \,$0.80	3.63$\,\pm \,$1.13
HMD-AP	7.50$\,\pm \,$2.41	24.99$\,\pm \,$9.19	70.09$\,\pm \,$5.27	21.59$\,\pm \,$5.61	2.39$\,\pm \,$1.22	1.58$\,\pm \,$0.57	2.44$\,\pm \,$1.62	3.80$\,\pm \,$1.43
HMD-ML	6.47$\,\pm \,$2.10	25.25$\,\pm \,$9.50	70.00$\,\pm \,$5.45	20.95$\,\pm \,$4.84	2.94$\,\pm \,$1.92	2.30$\,\pm \,$0.71	3.28$\,\pm \,$1.24	4.15$\,\pm \,$1.13
Post-VR	7.77$\,\pm \,$2.14	27.63$\,\pm \,$7.33	70.87$\,\pm \,$3.81	21.11$\,\pm \,$5.31	1.98$\,\pm \,$0.95	1.44$\,\pm \,$0.55	1.84$\,\pm \,$0.66	2.52$\,\pm \,$1.00

**Fig. 3:**
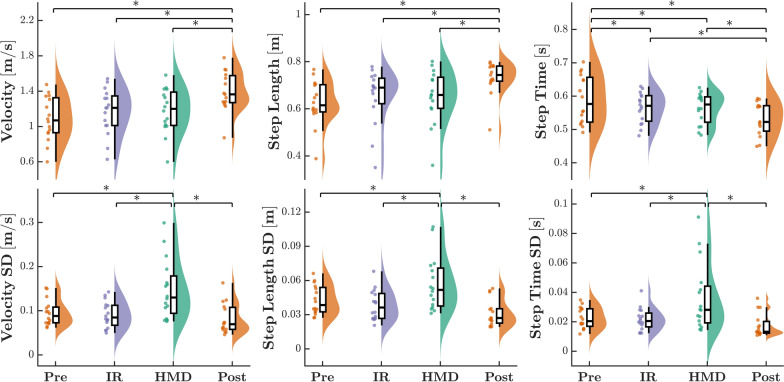
Mean and standard deviations for spatiotemporal gait parameters (*WS*, *SL*, *ST*) during unperturbed walking conditions. Trials without a dynamic virtual environment (*Pre-VR*, *Post-VR*), conducted in the IR, and while wearing the HMD are displayed in gold, purple, and green, respectively. Each dot displays the datapoint of a singular participant. Asterisks represent a significant difference (*p* < 0.05) between conditions, determined post-hoc using a Tukey’s HSD test

#### Statistics

Prior to calculating the measured parameters, we assessed each trial’s data for normality according to the Anderson–Darling test. The results indicated that a majority of the metrics followed a normal distribution. Consequently, we proceeded with calculating the mean and SD to describe the central tendencies and variability of the data respectively.

Analyses for all metrics were conducted utilizing linear mixed-effects models (LMM) (Tables 2 and 3). We performed distinct statistical evaluations for our two presented hypotheses regarding walking in VR under steady-state and challenged visual conditions. To address our first hypothesis, only experimental trials without visual perturbations were considered (baseline Pre-VR, isometric IR and HMD trials, and Post-VR). The corresponding condition was treated as a fixed model effect, and participant as a random effect. A single participant’s Post-VR trial was not included in this analysis due to a loss of motion capture data during the collection. Analyses for our second and third hypotheses regarding visual perturbations included all trials carried out within the virtual environment, but excluded the two trials without augmented optic flow (Pre-VR, Post-VR). VR display type (IR, HMD), perturbation type (ISO, AP and ML), and the interaction between the two were designated fixed model effects, and participant a random effect. For parameters displaying significant fixed effects, post-hoc comparisons were conducted via the Tukey’s Honest Significant Difference (HSD) test. Local effect sizes for significant fixed effects were reported using Cohen’s $f^2$ [44]. Statistical tests were performed in JMP Pro 16 (SAS Institute Inc., NC, USA) with the significance level set at 0.05.

## Results

**Fig. 4:**
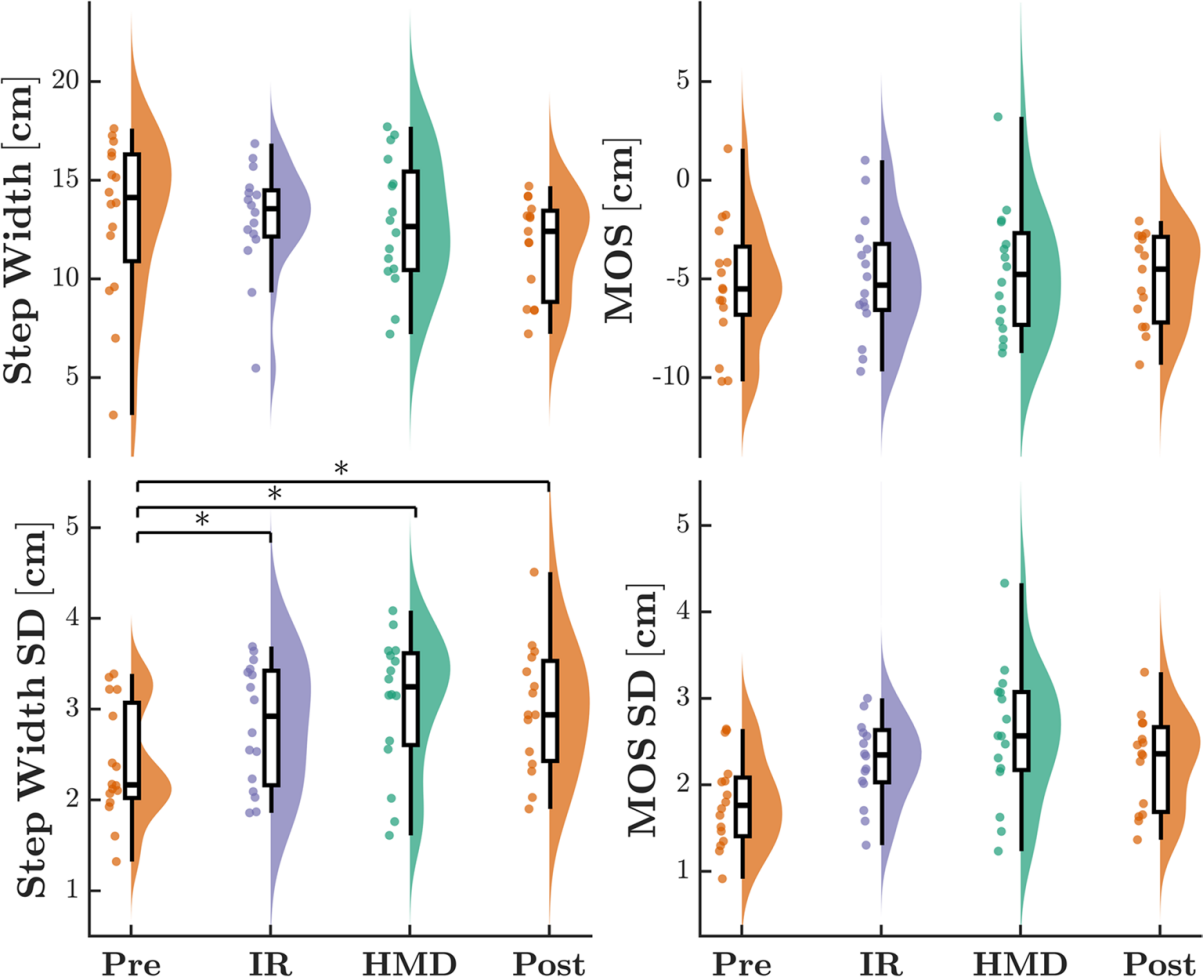
Mean and standard deviations for stability outcomes (*SW*, *MOS*) during unperturbed walking conditions. Trials without a dynamic virtual environment (*Pre-VR*, *Post-VR*), conducted in the IR, and while wearing the HMD are displayed in gold, purple, and green, respectively. Each dot displays the datapoint of a singular participant. Asterisks represent a significant difference (*p* < 0.05) between conditions, determined post-hoc using a Tukey’s HSD test

### Unperturbed locomotion

Significant main effects of condition were observed for all measured spatiotemporal parameters and their respective variabilities (Fig. 3). Participants walked significantly fastest during the Post-VR trial (WS: *F*_3,15_ = 12.61, *p* < 0.001, $f^2$ = 0.89) in comparison to all other trials. Higher Post-VR speed was governed by both a longer step length (SL: *F*_3,15_ = 9.84, *p* < 0.001, $f^2$ = 0.70) and shorter step time (ST: *F*_3,15_ = 12.62, *p* < 0.001, $f^2$ = 0.86). Participants also demonstrated longer step times in the baseline Pre-VR trial, although this did not result in a significantly slowed walking speed.

Between VR display types, no significant differences were found for mean values of WS, SL, and ST. However, walking in an HMD induced a significantly more variable velocity (WSV: *F*_3,15_ = 11.75, *p* < 0.001, $f^2$ = 0.88) than all other conditions, including IR. Speed variability increases were similarly characterized by increases in both SLV (SLV: *F*_3,15_ = 11.68, *p* < 0.001, $f^2$ = 0.86) and STV (STV: *F*_3,15_ = 9.36, *p* < 0.001, $f^2$ = 0.68).

**Fig. 5:**
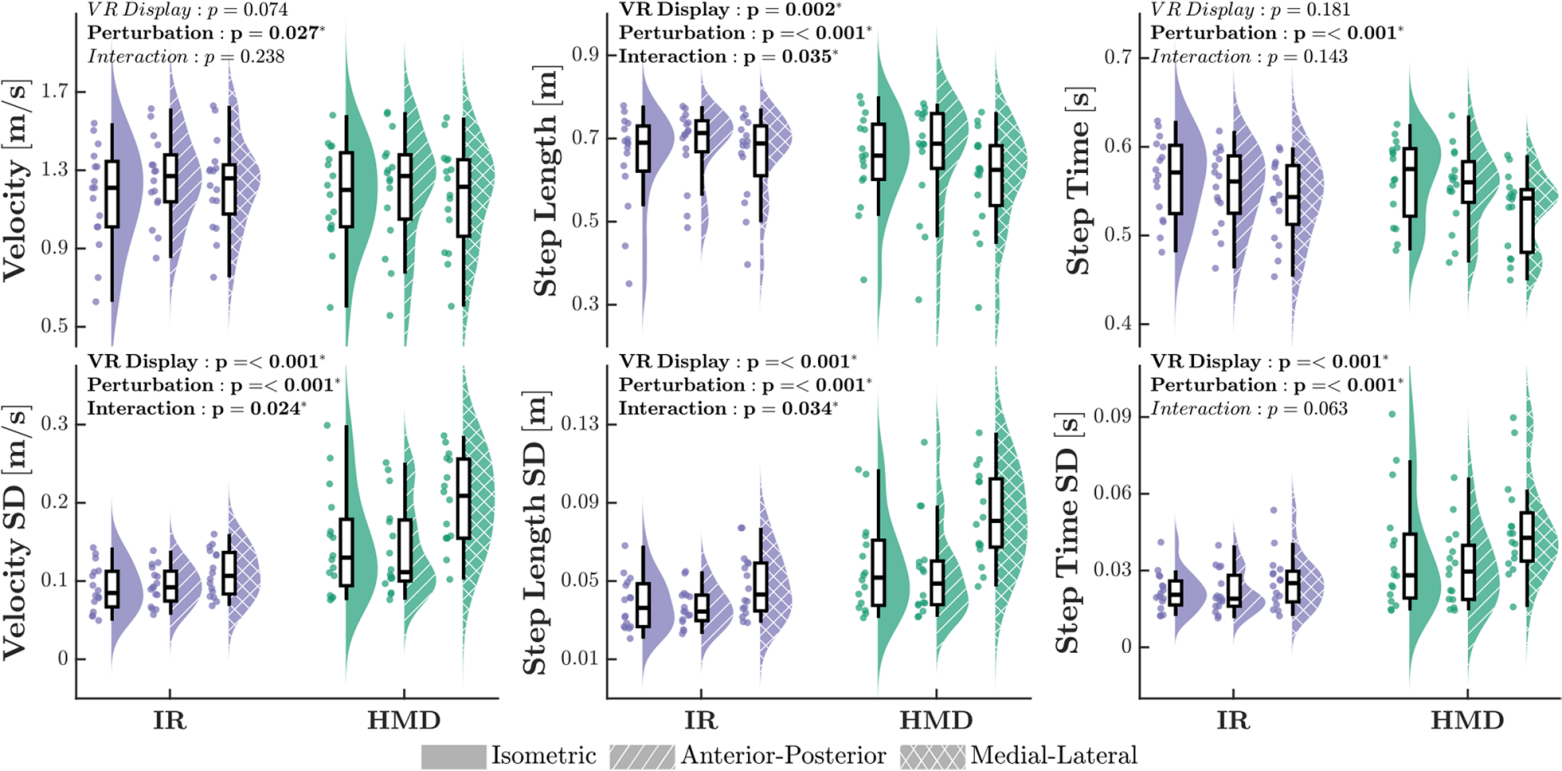
Mean and standard deviations for spatiotemporal gait parameters (*WS*, *SL*, *ST*) while navigating the virtual environment in the IR (purple) and HMD (green) systems. Trials featuring optic flow matched to the participant’s speed (*ISO*), perturbations parallel to forward motion (*AP*), and perturbations perpendicular to forward motion (*ML*) are represented with solid, striped, and cross-hatched violin plots, respectively. Each dot displays the datapoint of a singular participant. P-values for main effects and interactions are displayed on the plots, with significant values (p < 0.05) bolded, as determined by Tukey’s HSD test

Of the measured stability outcomes, a main effect of condition was observed only for SWV (Fig. 4). During the baseline Pre-VR trial, participants adopted a less variable SW pattern (SWV: *F*_3,15_ = 10.37, *p* < 0.001, $f^2$ = 0.73) compared to all other conditions. Post-hoc analyses revealed no significant differences in SWV between IR-ISO and HMD-ISO trials. There were no significant main effects of condition observed for any remaining stability outcomes (SW: *F*_3,15_ = 1.91, *p* = 0.142; MOS: *F*_3,15_ = 0.38, *p* = 0.770), although margin of stability variance approached significance (MOSV: *F*_3,15_ = 2.70, *p* = 0.057).

In-depth kinematic analysis revealed significant joint-level differences in the post-VR trial, namely for mean hip joint trajectory characteristics in the sagittal and frontal planes (Hip Flexion ROM: *F*_3,15_ = 8.62, *p*<.001, $f^2$ = 0.61; Peak Hip Flexion: *F*_3,15_ = 3.47, *p* = 0.024, $f^2$ = 0.23; Hip Adduction ROM: *F*_3,15_ = 8.35, *p*<.001, $f^2$ = 0.57) Participants exhibited increased hip flexion ROM in the post-VR condition compared to all other trials, and increased peak hip flexion compared to the baseline pre-VR trial. Further, variability measures for knee and ankle flexion trajectory characteristics were significantly increased in the HMD when compared to the Post-VR trial (Knee ROM SD: *F*_3,15_ = 5.10, *p* = 0.004, $f^2$ = 0.35; Ankle ROM SD:*F*_3,15_ = 3.33, *p* = 0.028, $f^2$ = 0.23; Ankle Peak SD: *F*_3,15_ = 4.63, *p* = 0.007, $f^2$ = 0.33).

**Fig. 6:**
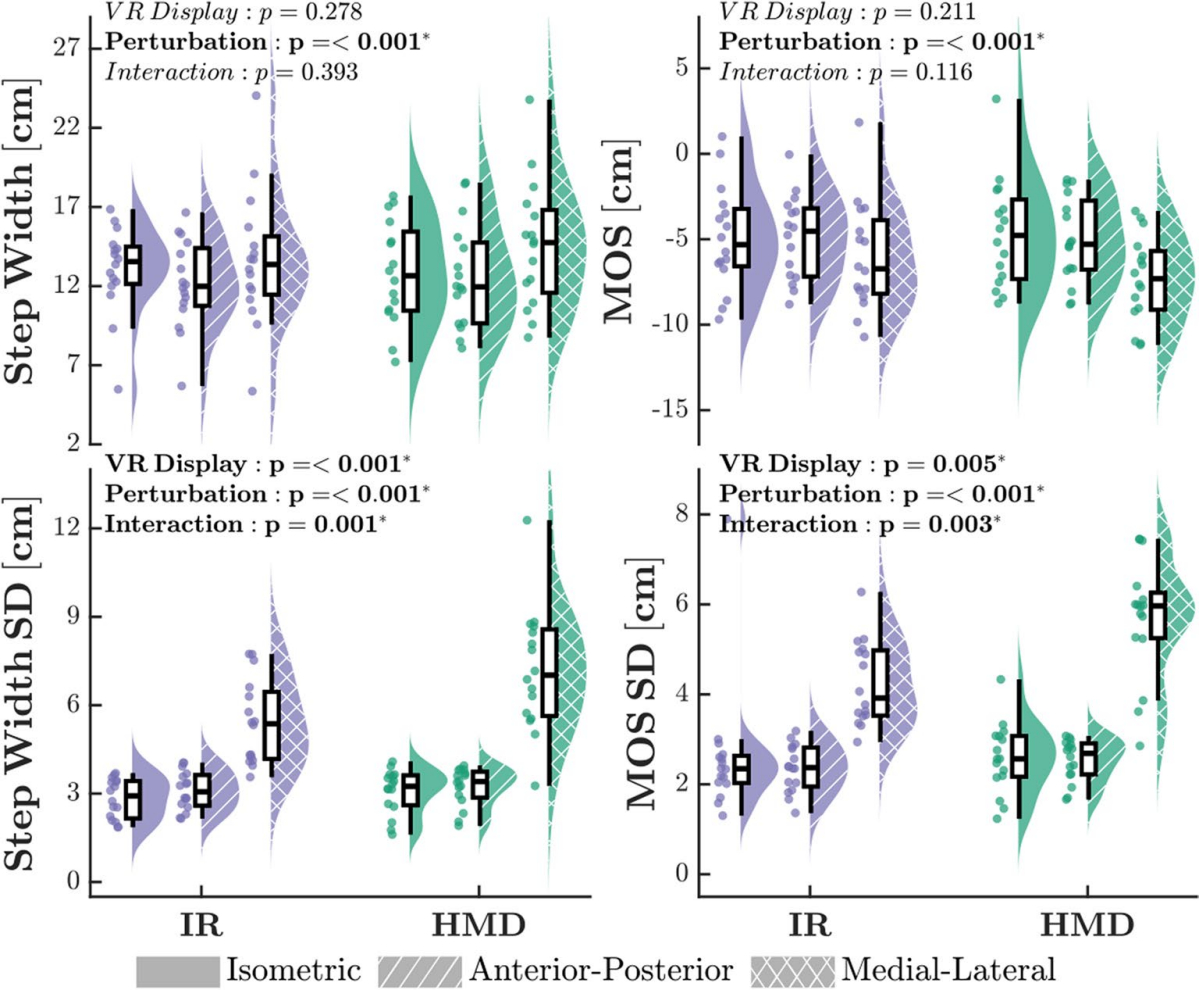
Mean and standard deviations for stability outcomes (*SW*, *MOS*) while navigating the virtual environment in the IR (purple) and HMD (green) systems. Trials featuring optic flow matched to the participant’s speed (*ISO*), perturbations parallel to forward motion (*AP*), and perturbations perpendicular to forward motion (*ML*) are represented with solid, striped, and cross-hatched violin plots, respectively. Each dot displays the datapoint of a singular participant. P-values for main effects and interactions are displayed on the plots, with significant values (p < 0.05) bolded, as determined by Tukey’s HSD test

### Optic flow perturbations

When considering all trials conducted in the virtual environment, we found that visual perturbation had a significant main effect on mean measures of walking speed (WS: *F*_2,15_ = 3.81, *p* = 0.027, $f^2$ = 0.14), step length (SL: *F*_2,15_ = 8.88, *p* < 0.001, $f^2$ = 0.33) and step time (ST: *F*_2,15_ = 25.71, *p* < 0.001, $f^2$ = 0.74) (Fig. 5). Participants walked faster when AP visual perturbations were present compared to velocity-matched optic flow (isometric), though neither was significantly different from the ML perturbation condition. ML perturbations resulted in a smaller overall step time compared to all other conditions, and shorter step length compared to AP. An additional main effect of VR display type (SL: *F*_2,15_ = 10.84, *p* = 0.002, $f^2$ = 0.24) and an interaction effect (SL: *F*_2,15_ = 3.50, *p* = 0.035, $f^2$ = 0.09) were present for mean step length. Although walking in the HMD elicited shorter step lengths than in the IR, this difference primarily reflects the HMD-ML condition, which had significantly smaller steps than all other factor combinations.

Significant main effects were observed for VR display type and visual perturbation across all measures of spatiotemporal variability, with additional interaction effects noted for WSV and SLV. Participants exhibited overall greater variability for WSV, SLV, and STV when viewing the scene in an HMD compared to an IR (WSV: *F*_1,15_ = 87.34, *p* < 0.001, $f^2$ = 1.34; SLV: *F*_1,15_ = 66.54, *p* < 0.001, $f^2$ = 1.04; STV: *F*_1,15_ = 63.74, *p* < 0.001, $f^2$ = 0.95). Additionally, introducing ML perturbations resulted in significantly higher variability measures than either the isometric optic flow or AP stimuli (WSV: *F*_2,15_ = 13.20, *p* < 0.001, $f^2$ = 0.47; SLV: *F*_2,15_ = 17.93, *p* < 0.001, $f^2$ = 0.59, STV: *F*_2,15_ = 9.33, *p* < 0.001, $f^2$ = 0.33). However, significant interaction effects (WSV: *F*_2,15_ = 3.92, *p* = 0.024, $f^2$ = 0.11; SLV: *F*_2,15_ = 3.53, *p* = 0.034, $f^2$ = 0.10) demonstrate that ML perturbations only elicited higher relative WSV and SLV responses if presented in the HMD, as no significant differences were observed across perturbation conditions in IR trials. A similar trend emerged for STV, however the interaction effect did not exceed significance (STV: *F*_2,15_ = 2.87, *p* = 0.063).

**Fig. 7:**
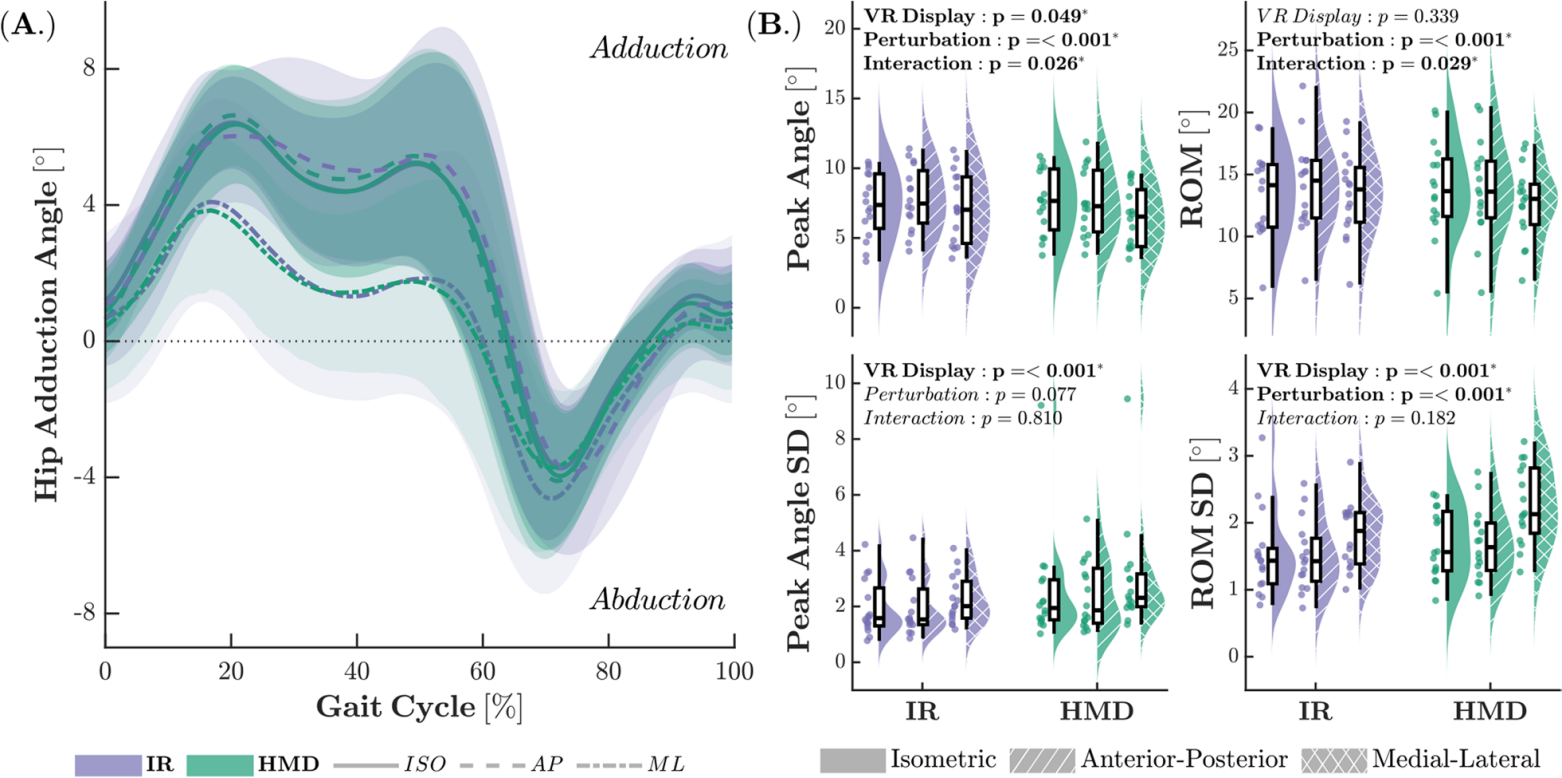
**A** Hip adduction angle trajectories for a representative participant. Angle values were normalized across the gait cycle each stride, with the mean trajectories indicated by solid lines and the shaded regions representing one standard deviation. **B** Mean and standard deviations for hip adduction angle kinematic measures (*Peak Angle*, *ROM*) while navigating the virtual environment in the IR (purple) and HMD (green) systems. Trials featuring optic flow matched to the participant’s speed (*ISO*), perturbations parallel to forward motion (*AP*), and perturbations perpendicular to forward motion (*ML*) are represented with solid, striped, and cross-hatched violin plots, respectively. Each dot displays the datapoint of a singular participant. P-values for main effects and interactions are displayed on the plots, with significant values (p < 0.05) bolded, as determined by Tukey’s HSD test

Regarding mean measures for stability metrics, a significant main effect of perturbation type was present in both SW and MOS (SW: *F*_2,15_ = 8.61, *p* < 0.001, $f^2$ = 0.26; MOS: *F*_2,15_ = 14.78, *p* < 0.001, $f^2$ = 0.46) (Fig. 6). Applying visual perturbations along the ML axis induced the greatest increase in mean step width, as well as the most destabilizing minimum margin of stability. We observed significant main effects on gait stability variability measures for VR display type (SWV: *F*_1,15_ = 13.08, *p* < 0.001, $f^2$ = 0.39; MOSV: *F*_1,15_ = 8.31, *p* = 0.005, $f^2$ = 0.30) and perturbation (SWV: *F*_1,15_ = 118.83, *p* < 0.001, $f^2$ = 4.01; MOSV: *F*_2,15_ = 78.18, *p* < 0.001, $f^2$ = 2.77), in addition to an interaction effect between the two (SWV: *F*_2,15_ = 7.22, *p* = 0.001, $f^2$ = 0.20; MOSV: *F*_2,15_ = 6.27, *p* = 0.003, $f^2$ = 0.18). Post-hoc analysis revealed significant SW and MOS variability increases in conditions containing ML perturbations for both VR types. Furthermore, variability for ML perturbation trials was significantly higher when presented in the HMD.

Statistical analysis revealed significant effects of VR type, perturbation, and their interaction on joint kinematics, particularly on peak angles, ROM, and their respective variabilities for hip adduction (Fig. 7). Across all conditions, the HMD-ML environment yielded the smallest peak adduction angles and ROM (Peak: *F*_1,15_ = 3.85, *p* = 0.026, $f^2$ = 0.10; ROM: *F*_1,15_ = 3.73, *p* = 0.029, $f^2$ = 0.10). Although no interaction effects were observed for variability measures, the HMD condition exhibited the highest variability in both peak angles and ROM (Peak SD: *F*_1,15_ = 12.66, *p* <.001, $f^2$ = 0.18; ROM SD: *F*_1,15_ = 14.04, *p* <.001, $f^2$ = 0.24), while the ML condition showed the greatest variability in ROM (*F*_1,15_ = 17.20, *p* <.001, $f^2$ = 0.52). Further details on the statistical analysis of kinematic data for all other joint angles are available in Additional File 1.

## Discussion

This study is among the first to directly compare the gait adaptations of self-paced treadmill users exposed to manipulated visual motion cues across different VR display modalities, namely IR and HMD systems. Novel to our approach, we investigated how sinusoidal visual perturbations—applied either in parallel with or perpendicular to forward motion—interact with specific systems to influence gait regulation and stability. Walking under isometrically matched virtual optic flow did not reveal significant differences in balance measures or average step measurements between the two VR systems, however, there was a notable increase in velocity variance when using the HMD. As anticipated, ML visual perturbations led to greater step variance and induced compensatory gait adaptations, such as shortened and widened steps. We further explored in greater detail how these gait adaptations differ between VR systems, particularly focusing on the unique disruptions to balance and velocity control observed in the HMD.

### Self-paced walking in VR systems

Our findings regarding unperturbed locomotion in HMD and IR virtual environments partially support our first hypothesis. While mean spatiotemporal gait parameter values for forward motion showed no significant differences between VR systems, the HMD condition was associated with a marked increase in variability. This indicates that when immersed in either virtual environment, participants were able to maintain their average walking speed patterns, in terms of similar mean step lengths and times, although their variability in sagittal step-to-step foot placement was greater in the HMD. In contrast, metrics for balance control in the lateral plane (i.e. SW and MOS) remained consistent for both mean and variability measures across trials, suggesting that stability in this direction was not significantly affected by the VR system under velocity-matched conditions.

There is currently no consensus regarding the impacts of VR augmentation on self-selected walking biomechanics, as findings within the literature have been inconsistent. Due to a wide variety of systems, environments, and experimental paradigms, studies have reported diverse outcomes, ranging from decreased walking speed compared to real-world conditions [25, 45] to increases [46, 47], or effects that are not considered clinically important [38, 48]. Despite the extensive use of IRs and HMDs in gait research and rehabilitation, direct comparisons between these technologies remain scarce, making it challenging to fully interpret and contextualize the variations across studies. A recent study by de Keersmaecker et al. [38] aimed to address this gap by investigating treadmill walking under varying optic flow gains using both IR and HMD systems. For healthy adults, they found no significant differences in walking speed, step time, step length, or step width when optic flow was velocity-matched between the two systems. This is consistent with our findings, which similarly demonstrated minimal changes in mean gait parameters across the VR conditions when no visual manipulations were present. However, our results highlight the additional importance of considering not only average gait performance, but also the underlying variability when detecting subtle adaptations to changing sensory information that may otherwise be overlooked.

### Post-VR walking speed adjustments

An unanticipated outcome of this study was the difference in self-selected walking speed observed between pre- and post-trial assessments when dynamic VR stimulation was removed. However, this outcome provided insights beyond our initial hypotheses. Participants exhibited faster walking speeds in the post-trial assessment that was collected following the completion of the series of VR trials. The walking speed was influenced by modifications in both step length and step time, though the post-trial condition remained identical to the pre-trial baseline condition. We considered whether this change could potentially be attributed to motor learning from prolonged practice using the SPT, prompting participants to adopt velocities that are more closely associated with overground walking patterns [46, 49]. However, previous research has shown that such variations typically stabilize after approximately 50–60 m of walking without VR stimulation [46]. Given the extended duration of our training periods, it is unlikely that learning effects alone account for the observed walking speed increase.

Alternatively, self-selected walking speeds in the post-VR trial may have shifted as a reflection of persisting internal model adjustments from processing the dynamic visual stimuli. Spatial relationships between visual motion and motor behaviors can be altered in a virtual environment using sensorimotor feedback mechanisms and subsequently carried over, leading to detectable changes in real-world motion [36, 50–53]. Specifically, individuals tend to underestimate their biomechanical walking speed after being recalibrated to a virtual environment in which augmented optic flow is velocity-matched [51]. Recalibration in the virtual environment, which creates a sense of slowed motion in the real-world, is a more likely explanation for why participants walked faster after VR exposure, as they attempted to align their adjusted perception of speed with the reduced optic flow density.

### Effects of visual perturbations across VR systems

While ML perturbations consistently elicited significant biomechanical effects, the relative magnitude of these responses varied depending on the VR system used to project the stimulus. In support of our third hypothesis, variability was increased for step width and MOS across ML trials, but these increases were significantly more pronounced in the HMD. Moreover, applying perturbations in the IR did not significantly impact step regulation, whereas, in the HMD, imposing ML perturbations led to increased variability in walking speed, step length and approached significance for step time. Kinematic analysis further showed that the ML perturbations resulted in the greatest changes to joint angle trajectories—most notably through decreased hip adduction and increased ankle ROM variability—contributing to the distinct gait patterns observed in this condition. To our knowledge, this study is the first to characterize directional differences in biomechanical responses to perturbations between VR systems, highlighting that system-specific factors can influence balance and velocity control under challenging visual conditions.

The biomechanical effects observed in the HMD system point to the potential for HMDs to provide a more sensitive environment for evaluating lateral gait stability, particularly under visual perturbations. Forward walking utilizes passively stable dynamics in the direction of motion, but requires active neural feedback to stabilize laterally through base of support and COM adjustments [15, 54]. Although the predictive nature of step width variability for fall prevention remains an open question, increasing experimental evidence has linked greater variability with gait instability [13, 55, 56]. Similarly, MOS variability, more so than mean values alone, has also been recognized as an effective method for capturing balance control, as it identifies the proportion of unstable steps taken over a series and the subsequent corrective actions required to avoid falling [57]. The increase in step width and MOS variability observed in this study suggests that participants reacted to stability challenges via step-to-step corrections in lateral foot placement under ML perturbations. This behavior is particularly pronounced in the HMD, which further amplified these dynamic responses.

Several possible explanations may illuminate the observed difference in step regulation strategies between the two VR systems. Although step width variability is primarily implicated for detecting gait instability, a relatively weak neuromechanical coupling has also been established between step width and step length variability [15, 58]. ML perturbations may have resulted in significant changes in step regulation for the HMD condition, whereas the IR condition did not, because the latter did not alter balance processes enough to impact step lengths to the same degree. An alternative, or perhaps further, reason for the change in step regulation could relate to the inherent redundancy between modulating step length and step time exploited to maintain a constant velocity [59]. In conditions with reduced optic flow information, individuals exhibit less step-to-step variability due to the decreased feedback from rapid visual changes that are required to stabilize their speed quickly and effectively [60]. The increased intensity of ML stimuli, which may have been further heightened by the HMD display, could have prompted participants to make more frequent corrections to their steps in order to maintain a stable velocity.

### Limitations and future directions

While we successfully identified divergent gait behaviors for each VR display modality, the experimental design does not allow for the isolation of system-specific features driving these observed changes, limiting our ability to pinpoint underlying mechanisms. One of the key factors potentially contributing to distinctions between the IR and HMD systems is the relative amount of visual information provided from the real-world environment. While wearing an HMD creates total separation from visual cues external to the device, the IR allows the user to see elements of the physical environment in their periphery, including the surrounding treadmill setup, boundaries of the visual display, and parts of their own body. Beyond providing additional information to internal models involved in motion perception [32, 35–37], it is also possible that real-world visual cues from the IR system specific to our setup—such as the treadmill belt and safety rails—may provide a visual anchor to help maintain an optimal position at the center of treadmill, augmenting users’ SPT performance. Preliminary findings from a previous pilot study indicate that participants adapt velocity patterns in response to the presence or absence of these treadmill elements in an HMD environment, suggesting that visual representations of the experimental setup play a role in directing locomotion [61]. Augmented reality (AR) headsets may offer a promising platform for manipulating the integration of real-world cues, by enabling users to simultaneously receive visual feedback from the virtual scene and physical environment even while immersed in an HMD. Future studies should continue to explore how particular visual and physical features that are inherent to each VR system influence gait behavior to disentangle the specific contributions of the visual surround to locomotor adaptations.

An additional limitation of this study is the absence of a direct measure of overground walking speed for each participant, which constrains the ability to fully contextualize treadmill-based speed outcomes in relation to real-world behaviors within our experimental paradigm. While previous studies have established a strong parallel between self-paced walking speeds overground and on a treadmill [46], the lack of overground walking speed data limits our capacity to independently verify whether the observed increase in treadmill walking speed after VR exposure reflects true recalibration effects, as hypothesized, or simply a return to baseline behaviors with training. Further, participants experienced the HMD and IR conditions in counterbalanced order, which introduces potential carryover effects that obfuscate the interpretation of the system specific influence on post-VR gait patterns. Future studies should incorporate overground walking speed measurements and consider experimental designs that more explicitly separate HMD and IR blocks to provide a more complete understanding of treadmill-based findings and further explore the relationship between VR exposure and real-world gait behaviors.

## Conclusions

Collectively, the present findings show that both the directionality of visual motion stimuli and the VR presentation modality play a role in shaping balance behaviors during locomotor visual perturbation paradigms. While the chosen VR display solely impacts walking speed control during unperturbed walking, introducing pseudorandom visual oscillations notably alters several key gait parameters between systems. Individuals are sensitive to visual perturbations misaligned with the direction of forward motion in both VR systems tested, though balance compensations are significantly elevated when wearing the HMD. Further, room-based systems do not provide effectual visual stimulation for inducing changes to walking speed control that were present at similar perturbation magnitudes in the HMD, as indicated by a lack of response across trials. In summary, our results demonstrate that individuals adopt the most pronounced gait pattern adaptations while ambulating in an HMD, which can be heightened further by coupling continuous visual perturbations along the ML axis. Broadly, this implies that HMDs can be harnessed to investigate gait stability during sensory conflict, potentially offering a more effective and practical alternative to immersive room setups.

## Supplementary Information

Supplementary material 1.

## Data Availability

The datasets used and analyzed during the current study are available from the corresponding author, W.G.W, on reasonable request.

## Abbreviations

AP: Anterior–posterior
BOS: Base of support
COM: Center of mass
HMD: Head-mounted display
HSD: Honest significant difference
IR: Immersive room
ISO: Isometric
LMM: Linear mixed-effects models
ML: Medio-lateral
MOS: Margin of stability
ROM: Range of motion
SD: Standard deviation
SL: Step length
SP: Step time
SPT: Self-paced treadmill
SW: Step width
VR: Virtual reality
WS: Walking speed
XCOM: Extrapolated center of mass
